# Effect of pegylated interferon α-2b on low-density lipoprotein in patients with chronic hepatitis B

**DOI:** 10.3389/fmed.2026.1787001

**Published:** 2026-07-03

**Authors:** Baiguo Xu, Aili Fan, Baoxin Qian, Jing Liang, Tinghong Li, Weili Yin, Huiling Xiang, Qing Ye

**Affiliations:** 1 Tianjin Third Central Hospital, Tianjin, China; 2 Central Hospital, Tianjin University, Tianjin, China; 3 Tianjin Institute of Hepatobiliary Disease, Tianjin, China; 4 Tianjin Key Laboratory of Extracorporeal Life Support for Critical Diseases, Tianjin, China; 5 Artificial Cell Engineering Technology Research Center, Tianjin, China; 6 The Third Central Clinical College of NanKai University, Tianjin, China

**Keywords:** antiviral therapy, chronic hepatitis B, low-density lipoprotein, pegylated interferon α-2b, serum lipoprotein

## Abstract

**Background:**

The effect of pegylated interferon α-2b (Peg-IFNalpha-2b) on lipid metabolism in chronic hepatitis B (CHB) patients remains controversial. This study aimed to investigate the dynamic changes of low-density lipoprotein (LDL) levels during different antiviral regimens and explore their potential clinical significance.

**Methods:**

A total of 585 CHB patients were enrolled in this real-world cohort study and divided into three groups: nucleos(t)ide analogs (NAs) monotherapy group (*n* = 96), NAs + Peg-IFNalpha-2b combination group (*n* = 396), and Peg-IFNalpha-2b monotherapy group (*n* = 93). Serum lipid levels were monitored at baseline and weeks 12, 24, 36, and 48. Generalized additive mixed models were used to adjust for key confounders including sex, age, body mass index (BMI), fatty liver status, cirrhosis, HBV DNA, HBsAg, HBeAg and so on.

**Results:**

After 48 weeks of treatment, LDL levels decreased by 0.3–0.4 mmol/L in the Peg-IFNalpha-2b monotherapy group compared with baseline, with an intermediate reduction in the combination group and no significant change in the NAs monotherapy group. The association remained significant after adjusting for key confounders (*P* < 0.05), with consistent results across subgroups such as age, gender and fatty liver status.

**Conclusion:**

Peg-IFNalpha-2b treatment significantly reduces LDL levels in CHB patients. This mild but consistent LDL reduction may bring potential long-term cardiovascular and hepatic benefits, especially for patients with comorbid metabolic disorders.

## 1 Introduction

Chronic hepatitis B (CHB) poses a serious threat to global health, with an estimated 257 million people worldwide living with chronic HBV infection and approximately 887,000 deaths annually from HBV-related diseases (1). Antiviral therapy is the cornerstone of CHB management, effectively suppressing viral replication and delaying disease progression (2). Pegylated interferon α-2b (Peg-IFNalpha-2b), an important antiviral agent, exerts not only direct antiviral activity but also immunomodulatory effects (3). Nevertheless, Peg-IFNalpha-2b therapy may induce various adverse effects, among which its influence on lipid metabolism has attracted increasing attention (4). Dyslipidemia is closely associated with cardiovascular diseases, and low-density lipoproteins (LDL), as a major component of blood lipids, play a critical role in cardiovascular risk assessment (5). Previous studies have reported that interferon therapy may alter lipid levels, but the results have been inconsistent (6, 7). At present, large-scale and systematic clinical studies investigating the effect of Peg-IFNalpha-2b on LDL in CHB patients remain lacking. Therefore, this study aimed to explore the impact of Peg-IFNalpha-2b on serum LDL levels in CHB patients and to analyze the relationship between different antiviral regimens and LDL changes, thereby providing valuable insights for clinical practice.

## 2 Materials and methods

### 2.1 Study design

This was a prospective cohort study conducted at Tianjin University Central Hospital from November 2020 to May 2024 [Clinical Trial Registration: ChiCTR2000028881 [Chinese Clinical Trial Registry)]. This prospective cohort study was designed with a clear primary endpoint (change in serum LDL from baseline to week 48, ΔLDL48) and secondary endpoints (longitudinal changes in lipid profiles, HBsAg clearance rate at week 48). Sample size was calculated for the primary endpoint of ΔLDL48. We assumed a between-group difference of 0.3 mmol/L in LDL reduction, with a standard deviation of 0.75 mmol/L (based on published Peg-IFNα-2b lipid studies). Using a two-sided α of 0.05 and 80% statistical power, the required sample size was 88 patients per group. To account for 5% dropout, we should enroll a minimum of 93 patients per group. The study protocol was approved by the Ethics Committee of Third Central Hospital of Tianjin prior to patient enrollment, and all participants provided written informed consent. This prospective cohort study collected clinical data of eligible CHB patients and analyzed the effects of different antiviral regimens on serum LDL levels.

### 2.2 Study population

**Figure 1:**
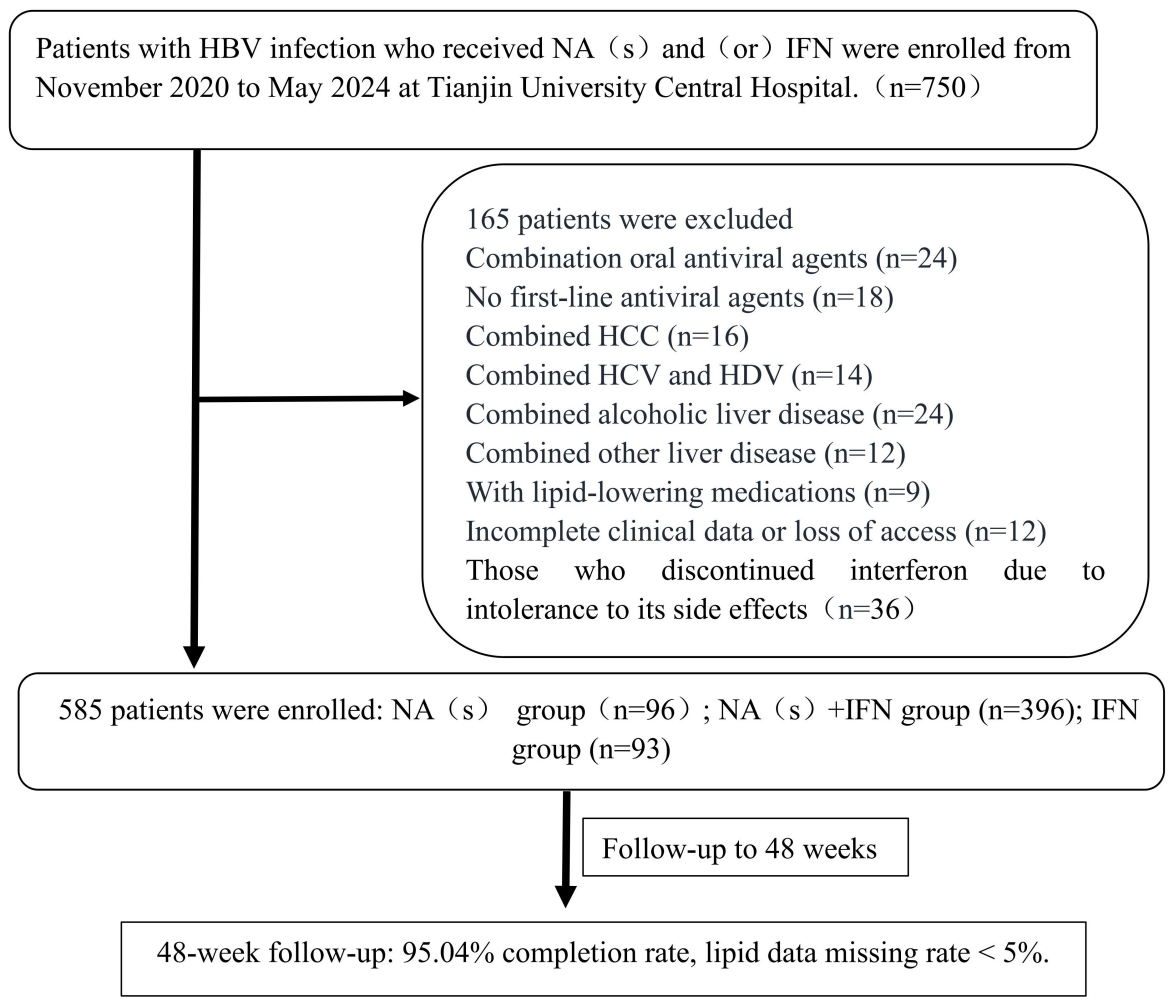
Flowchart of patient enrollment in the prospective cohort study. From November 2020 to May 2024, a total of 750 patients with chronic hepatitis B (CHB) receiving nucleos(t)ide analogs [NAs] and/or pegylated interferonalpha-2b (Peg-IFNalpha-2b) were initially screened at Tianjin University Central Hospital. Among them, 165 patients were excluded. Finally, 585 eligible patients were enrolled and assigned to three groups: NAs monotherapy group (*n*=96), NAs + Peg-IFNalpha-2b combination therapy group (*n*=396), and Peg-IFNalpha-2b monotherapy group (*n*=93). Follow-up completion rates were 100% at baseline, 98.12% at week 12, 97.44% at week 24, 96.58% at week 36, and 95.04% at week 48. Corresponding data missing rates were 0, 1.88, 2.56, 3.42, and 4.96%, respectively.

CHB patients treated at the Department of Gastroenterology and Hepatology, Tianjin University Central Hospital, between November 1, 2020, and May 1, 2024, were screened. Inclusion criteria: (1) Met the diagnostic criteria for CHB (8); (2) Treated with first-line nucleos(t)ide analogs [NAs: entecavir (ETV), tenofovir disoproxil fumarate (TDF), or tenofovir alafenamide fumarate (TAF)] and/or Peg-IFNalpha-2b injection [Including both treatment-naïve and nucleos(t)ide] analog (NAs)-experienced patients. Treatment-naïve patients: no prior history of any NAs or interferon antiviral treatment; NA-experienced patients: received at least 6 months of NAs monotherapy before enrollment, with no change in treatment regimen for ≥6 months before enrollment; (3) Age between 20 and 70 years; (4) All patients in the NAs + Peg-IFNalpha-2b group, with HBsAg ≤ 5,000 IU/ml and sustained suppression of HBV DNA [defined as: serum HBV DNA < 20 IU/ml (high-sensitivity quantitative PCR assay, lower limit of detection: 10 IU/ml) for at least six consecutive months, with at least three consecutive negative tests (interval ≥ 1 month between tests)]. Exclusion criteria: (1) Use of other non–first-line oral antivirals or combination therapy involving non–first-line agents during the study period. (2) Concomitant use of lipid-lowering therapy (western medicine and/or traditional Chinese medicine). (3) Coexistence of other liver diseases (including alcoholic liver disease, HCV or HDV infection, autoimmune hepatitis, and primary biliary cholangitis) or pregnancy. (4) Presence of hepatocellular carcinoma or other malignancies. (5) Decompensated HBV-related cirrhosis (9). (6) Treatment discontinuation due to intolerance to Peg-IFNalpha-2b. The detailed flowchart of patient enrollment is presented in Figure 1.

### 2.3 Data collection and laboratory testing

Baseline demographic and clinical data were collected, including age, sex, height, weight, cirrhosis status, treatment history, family history of HBV, and fatty liver status. Body mass index (BMI) was calculated. Laboratory data included complete blood count, liver function tests, lipid profile, alpha-fetoprotein (AFP), HBV serological markers, and HBV DNA quantification. Lipid parameters included total cholesterol (TC), triglycerides (TG), high-density lipoprotein (HDL), and LDL cholesterol, measured with an automated biochemical analyzer. HBV serological markers were detected by chemiluminescence immunoassay, while HBV DNA levels were measured using real-time quantitative PCR, with a detection limit of 20 IU/ml.

Changes from baseline at different time points were defined as follows: for example, DTC120 represented the difference between week 12 TC and baseline TC; DTG120 represented the difference between week 12 TG and baseline TG; similarly, ΔLDL48 represented the difference between week 48 LDL and baseline LDL.

MASLD was diagnosed according to the Guidelines for the Diagnosis and Treatment of Non-Alcoholic Fatty Liver Disease (2024 Edition, China) (10): ultrasonic features of hepatic steatosis (hepatic parenchymal echo enhancement, posterior acoustic attenuation, unclear intrahepatic vascular boundaries). What's more, it is necessary to rule out excessive alcohol consumption (for men, weekly ethanol intake ≥ 210 g, for women, ≥ 140 g) and other causes that can lead to fatty liver, and the patient must have at least one component of metabolic syndrome (MetS): overweight/obesity; elevated arterial blood pressure/hypertension; pre-diabetes or type 2 diabetes; elevated blood triglycerides; decreased high-density lipoprotein cholesterol.

### 2.4 Treatment regimens and procedures

Patients were divided into three groups according to their treatment regimens: (1) NAs group: oral monotherapy with one first-line NAs: ETV 0.5 mg/day, TDF 300 mg/day, or TAF 25 mg/day. (2) NAs + Peg-IFNalpha-2b group: combination therapy of oral first-line NAs with Peg-IFNalpha-2b (Pegbing^®^, Xiamen Amoytop Biotech Co., Ltd.) injection, 180 μg once weekly, administered subcutaneously. (3) Peg-IFNalpha-2b group: Peg-IFNalpha-2b monotherapy, same dosage as above (11).

### 2.5 Follow-up procedures

Patients were followed up every 12 weeks for a total of 48 weeks, during which each follow-up included medical history inquiry, physical examination, laboratory tests (TC, TG, HDL, LDL), and medication adherence assessment. Adverse events and treatment compliance were documented. All patients also received routine counseling and education on healthy diet and lifestyle. All blood samples were collected after 8–12 h overnight fasting at each time point (baseline, weeks 12, 24, 36, 48). Lipid profiles were tested in the same clinical laboratory with unified reagents and quality control to ensure consistency. Missing lipid profile data (overall < 5% across all time points) were handled using multiple imputation, implemented via the mice package in R. The imputation model included all baseline covariates (age, sex, BMI, treatment group, baseline LDL, ALT, AST, HBsAg, HBV DNA) and all observed follow-up lipid values. Imputation was performed under the missing at random (MAR) assumption, which is appropriate for this study as missingness was only related to observed follow-up visit patterns. Convergence was verified via trace plots of imputed variables across 20 iterations, with no evidence of non-convergence. Five imputed datasets were generated, and results were pooled using Rubin's rules to account for imputation uncertainty. Sensitivity analysis using complete-case analysis confirmed the robustness of all primary findings. Follow-up completion rates and data missing rates at each time point were as follows: baseline (100% completion, 0% missing), week 12 (98.12% completion, 1.88% missing), week 24 (97.44% completion, 2.56% missing), week 36 (96.58% completion, 3.42% missing), and week 48 (95.04% completion, 4.96% missing). The overall missing rate for lipid profile data was less than 5% throughout the study period.

### 2.6 Statistical analysis

All statistical analyses were performed using SPSS version 25.0 and R software. Missing lipid profile data at follow-up time points (accounting for < 5% of total data) were handled using multiple imputation with five imputed datasets. Sensitivity analysis using complete case analysis confirmed the robustness of the results. Continuous variables were expressed as mean ± standard deviation (x ± s), and compared between groups using analysis of variance (ANOVA) or *t*-test. Categorical variables were presented as counts (*n*) and percentages (%), with intergroup comparisons performed using the χ^2^ test. Univariate analyses were first conducted to identify factors associated with serum ΔLDL48. Generalized additive mixed models (GAMM) were used to analyze the association between treatment regimens and ΔLDL48, with thin-plate regression splines for continuous covariates to capture potential non-linear relationships. The model included random intercepts for each patient to account for within-subject correlation in repeated measurements. All smooth terms were specified with a basis dimension (k) of 8, and convergence was verified via residual diagnostics. All analyses were implemented using the mgcv package in R (version 4.3.1). Confounders were selected based on a pre-specified principle: all variables with *P* < 0.05 in univariate analysis of ΔLDL48 were included in the multivariable model and the indicators that are considered meaningful in clinical practice. Stratified analyses were additionally performed to clarify the effect of treatment regimens on serum ΔLDL48. A *P*-value < 0.05 was considered statistically significant. For pairwise comparisons among the three groups, Bonferroni correction was applied, and *P* < 0.017 was considered statistically significant. For multiple comparisons among the three treatment groups, Bonferroni *post-hoc* test was performed to determine pairwise differences, and *P* < 0.017 was considered statistically significant.

## 3 Results

### 3.1 Baseline clinical and demographic characteristics of patients receiving different antiviral regimens

A total of 585 CHB patients were enrolled, including 96 in the NAs group, 396 in the Peg-IFNalpha-2b + NAs group, and 93 in the Peg-IFNalpha-2b monotherapy group. The mean age was 42.77 ± 9.16 years, and 73.04% were male. The overall MASLD prevalence was 58.63%, which is consistent with the high incidence of MASLD in overweight populations (BMI ≥ 24 kg/m^2^) in China (10). Patients in the NAs group were significantly older (45.2 ± 8.7 vs. 42.1 ± 9.2 years, *P* < 0.05). Compared with the NAs group, patients receiving Peg-IFNalpha-2b had higher baseline LDL and HBV DNA levels (*P* < 0.05). However, the incidence of non-alcoholic fatty liver disease (MASLD; NAs group 67.71% vs. Peg-IFNalpha-2b + NAs group 55.05% vs. Peg-IFNalpha-2b group 53.76%, *P* = 0.066) and body mass index (BMI; NAs group 25.03 ± 3.58 vs. Peg-IFNalpha-2b + NAs group 25.49 ± 3.70 vs. Peg-IFNalpha-2b group 25.03 ± 3.50, *P* = 0.375) did not differ significantly among the groups. Notably, compared with Peg-IFNalpha-2b, the NAs group had significantly higher HBsAg levels (log_10_ IU/ml: 3.16 ± 0.77 vs. 2.26 ± 1.43, *P* < 0.001) and a higher prevalence of cirrhosis (25.29 vs. 9.88%, *P* = 0.035; Table 1).

**Table 1:** Demographic and clinical characteristics of participants at baseline.

Variables	NAs	NAs + Peg-IFNalpha-2b	Peg-IFNalpha-2b	*P*-value
*N*	96	396	93	
Age (years)	45.50 ± 10.16	42.16 ± 8.78	42.30 ± 9.18	0.005
Gender, *n* (%)				0.035
Male	67 (69.79%)	302 (76.27%)	59 (63.44%)	
Female	29 (30.21%)	94 (23.73%)	34 (36.56%)	
Waistline (cm)	87.89 ± 9.51	89.61 ± 10.67	87.15 ± 10.10	0.080
Height (cm)	1.70 ± 0.09	1.71 ± 0.08	1.69 ± 0.08	0.051
Weight (kg)	72.87 ± 13.29	75.01 ± 13.72	71.65 ± 13.71	0.072
BMI (kg/m^2^)	25.03 ± 3.58	25.49 ± 3.70	25.03 ± 3.50	0.375
CHB family history, *n* (%)				0.347
Yes	48 (50.00%)	224 (56.57%)	52 (55.59%)	
No	48 (50.00%)	172 (43.43%)	41 (44.41%)	
With other diseases				0.877
Yes	14 (14.58%)	56 (14.14%)	16 (17.02%)	
No	82 (85.42%)	340 (85.86%)	77 (82.98%)	
Pr-treatment history				< 0.001
Yes	81 (84.38%)	225 (56.82%)	10 (10.75%)	
No	15 (15.62%)	171 (43.18%)	83 (89.25%)	
NAs treatment				0.122
ETV	48 (50.00%)	175 (44.19%)		
TDF	29 (30.21%)	135 (34.09%)		
TAF	19 (19.79%)	86 (21.72%)		
Fatty liver, *n* (%)				0.066
Yes	65 (67.71%)	218 (55.05%)	50 (53.76%)	
No	31 (32.29%)	178 (44.95%)	43 (46.24%)	
Cirrhosis				0.035
Yes	22 (25.29%)	60 (19.35%)	8 (9.88%)	
No	65 (74.71%)	250 (80.65%)	73 (90.12%)	
HBV DNA (log_10_ IU/ml)	1.70 (1.70–2.70)	1.78 (1.70–3.42)	2.48 (1.94–3.46)	< 0.001
HBV DNA				< 0.001
Positive	23 (23.96%)	173 (43.69%)	69 (74.19%)	
Negative	73 (76.04%)	223 (56.31%)	24 (25.81%)	
HBsAg (log_10_ IU/ml)	3.16 ± 0.77	3.01 ± 1.08	2.26 ± 1.43	< 0.001
HBeAg				0.098
Positive	36 (37.50%)	109 (27.53%)	16 (17.20%)	
Negative	60 (62.50%)	287 (72.47%)	77 (82.80%)	
ALT (U/L)	25.00 (18.00–35.00)	27.00 (18.00–45.00)	24.00 (18.00–46.25)	0.387
AST (U/L)	22.00 (18.00–28.25)	23.00 (19.00–32.00)	22.00 (18.00–31.00)	0.524
TBIL (μmol/L)	18.24 ± 8.42	16.75 ± 6.64	15.61 ± 6.00	0.035
ALP (U/L)	80.76 ± 24.30	74.55 ± 20.95	66.30 ± 15.70	< 0.001
GGT (U/L)	22.50 (17.00–33.50)	24.00 (16.00–39.00)	23.00 (14.00–34.00)	0.490
AFP (ng/ml)	2.80 (2.08–3.77)	2.70 (2.01–3.62)	2.73 (2.00–4.20)	0.624
TC (mmol/L)	4.21 (3.90–4.85)	4.62 (3.88–5.20)	4.62 (3.96–5.08)	0.104
TG (mmol/L)	1.05 (0.81–1.71)	1.15 (0.84–1.61)	1.16 (0.85–1.79)	0.899
HDL (mmol/L)	1.04 ± 0.21	1.12 ± 0.29	1.14 ± 0.23	0.123
LDL (mmol/L)	2.49 ± 0.58	2.79 ± 0.76	2.81 ± 0.75	0.038
TC at 12 week (mmol/L)	4.55 ± 1.25	4.12 ± 2.08	3.95 ± 0.44	0.652
TG at 12 week (mmol/L)	1.52 (0.95–3.83)	1.52 (1.06–2.00)	1.40 (1.03–1.86)	0.758
HDL at 12 week (mmol/L)	0.92 ± 0.25	0.95 ± 0.31	0.99 ± 0.22	0.847
LDL at 12 week (mmol/L)	2.05 ± 1.27	2.16 ± 0.64	2.23 ± 0.46	0.804
TC at 24 week (mmol/L)	4.25 ± 1.16	4.12 ± 2.01	4.20 ± 1.22	0.927
TG at 24 week (mmol/L)	1.33 (0.80–1.94)	1.52 (1.15–2.05)	1.34 (1.06–2.10)	0.049
HDL at 24 week (mmol/L)	1.02 ± 0.26	0.91 ± 0.22	1.00 ± 0.27	0.013
LDL at 24 week (mmol/L)	2.45 ± 0.88	2.12 ± 0.68	2.12 ± 0.79	0.189
TC at 36 week (mmol/L)	4.28 ± 0.95	3.99 ± 0.73	4.27 ± 0.99	0.259
TG at 36 week (mmol/L)	1.29 ± 0.59	1.53 ± 0.68	1.63 ± 1.17	0.341
HDL at 36 week (mmol/L)	1.06 ± 0.21	0.93 ± 0.18	0.95 ± 0.17	0.532
LDL at 36 week (mmol/L)	2.49 ± 0.89	2.23 ± 0.55	2.43 ± 0.83	0.253
TC at 48 week (mmol/L)	4.33 ± 0.93	4.07 ± 0.78	4.08 ± 0.91	0.209
TG at 48 week (mmol/L)	1.21 (0.81–1.86)	1.40 (0.98–1.99)	1.49 (1.15–2.58)	0.253
HDL at 48 week (mmol/L)	1.10 ± 0.44	0.97 ± 0.24	0.97 ± 0.27	0.058
LDL at 48 week (mmol/L)	2.64 ± 0.81	2.29 ± 0.71	2.25 ± 0.65	0.033

Results in the table are presented as mean ± SD or Median (Q1-Q3) for quantitative data, and as N (%) for categorical data. Continuous variables were analyzed using the Kruskal–Wallis rank sum test.

For categorical variables with expected counts < 10, Fisher's exact test was applied.

NAs, nucleoside (nucleotide) analogs; CHB, chronic hepatitis B; HBV DNA, hepatitis B virus deoxyribonucleic acid; HBsAg, hepatitis B surface antigen; HBeAg, hepatitis B e antigen; ALT, alanine aminotransferase; AST, aspartate aminotransferase; TBIL, total bilirubin; ALP, alkaline phosphatase; GGT, gamma-glutamyl transferase; AFP, alpha-fetoprotein; TC, total cholesterol; TG, triglycerides; HDL, high-density lipoprotein; LDL, low-density lipoprotein.

### 3.2 Changes in lipid levels compared with baseline

**Figure 2:**
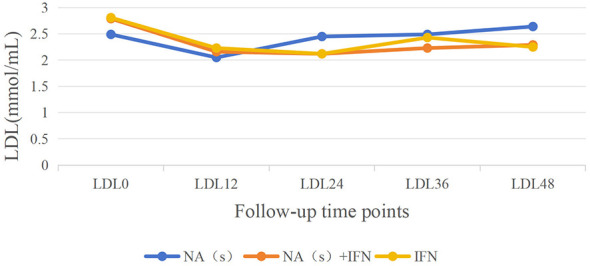
Dynamic changes in serum low-density lipoprotein (LDL) levels across different antiviral regimens in CHB patients. This line graph depicts the dynamic changes in the mean LDL levels of the three groups (NAs, NAs + Peg-IFNalpha-2b, Peg-IFNalpha-2b) at baseline (LDL0) and follow-up weeks 12 (LDL12), 24 (LDL24), 36 (LDL36), and 48 (LDL48). During follow-up, the mean LDL levels in the NAs group fluctuated slightly (2.49 → 2.05 → 2.45 → 2.49 → 2.64 mmol/L), while the NAs + Peg-IFNalpha-2b group (2.79 → 2.16 → 2.12 → 2.23 → 2.29 mmol/L) and Peg-IFNalpha-2b group (2.81 → 2.23 → 2.12 → 2.43 → 2.25 mmol/L) showed a sustained downward or stable trend, with LDL levels remaining lower than the NAs group from week 12 onwards.

**Figure 3:**
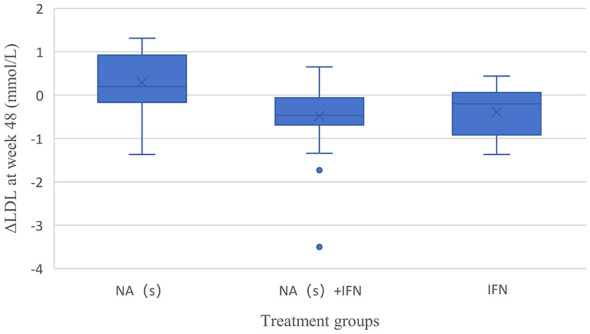
Box plots of 48-week changes in serum LDL (ΔLDL48) across different antiviral regimens. This box plot compares ΔLDL48 (the difference between serum LDL levels at week 48 and baseline) among the three treatment groups (NAs monotherapy, NAs + Peg-IFNalpha-2b, Peg-IFNalpha-2b monotherapy). The horizontal axis represents the treatment groups, and the vertical axis represents ΔLDL48 values. Statistical analysis showed a significant difference in ΔLDL48 among the three groups (*P* < 0.001). The NAs group had a higher ΔLDL48 (indicating less LDL reduction or even elevation), while the NAs + Peg-IFNalpha-2b and Peg-IFNalpha-2b groups had lower ΔLDL48, confirming that Peg-IFNα-2b-containing regimens significantly reduced LDL levels compared with NAs monotherapy after 48 weeks of treatment.

**Figure 4:**
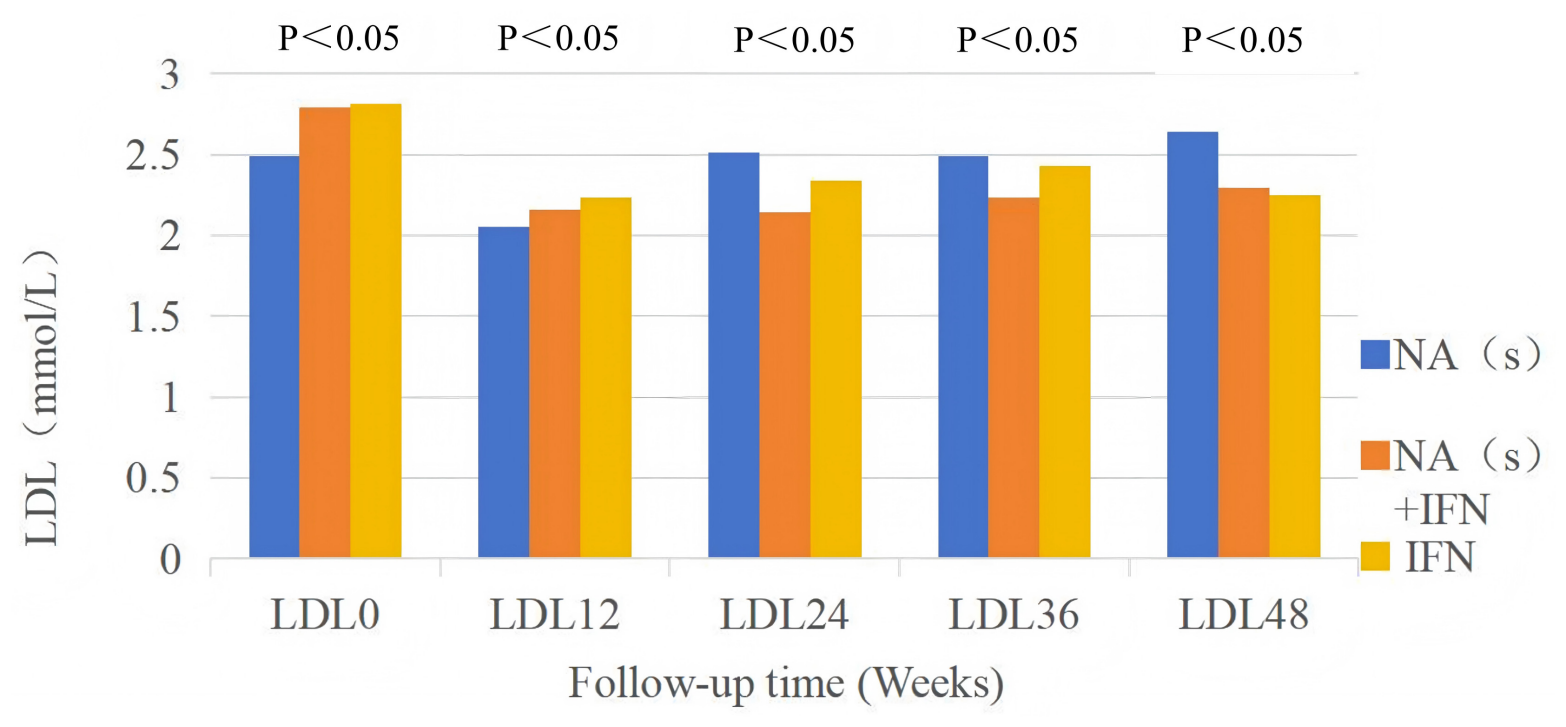
Comparison of mean serum LDL levels at different follow-up time points across antiviral regimens. This graph presents the mean serum LDL levels (unit: mmol/L) of CHB patients in the three treatment groups (NAs monotherapy, NAs + Peg-IFNalpha-2b, Peg-IFNalpha-2b monotherapy) at baseline (LDL0) and follow-up weeks 12 (LDL12), 24 (LDL24), 36 (LDL36), and 48 (LDL48).

Relative to baseline, lipid levels showed varying degrees of change across the three groups at different time points (Table 2). Except for TG, patients receiving Peg-IFNalpha-2b exhibited a downward trend in TC, HDL, and LDL at multiple time points. During 48 weeks of follow-up, serum LDL levels in the Peg-IFNalpha-2b group showed an overall downward trend, decreasing from 2.81 ± 0.75 mmol/L at baseline to 2.25 ± 0.65 mmol/L at week 48, with slight fluctuations between weeks 12 and 36 (Figure 2). At week 48, LDL480 was significantly reduced compared with the NAs group (*P* < 0.05; Figures 3, 4).

**Table 2:** Differences in lipid profiles between various time points and baseline.

Variables	NAs	NAs + Peg-IFNalpha-2b	Peg-IFNalpha-2b	*P*-value
*N*	96	396	93	
DTC120	−0.41 (−0.68 to 0.02)	−0.42 (−0.91 to −0.15)	−0.15 (−0.72 to−0.04)	0.784
DTG120	0.35 (−0.44 to 2.59)	0.35 (0.11 to 0.80)	0.38 (0.17 to 1.2)	0.018
DHDL120	−0.13 (−0.36 to 0.06)	−0.16 (−0.30 to −0.03)	−0.12 (−0.24 to −0.08)	0.919
DLDL120	−0.52 (−1.00 to −0.01)	−0.60 (−0.92 to −0.26)	−0.28 (−0.71 to −0.14)	0.793
DTC240	−0.47 (−0.88 to 0.32)	−0.54 (−0.97 to −0.10)	−0.45 (−0.67 to −0.37)	0.935
DTG240	0.04 (−0.34 to 0.28)	0.30 (0.03 to 0.77)	0.46 (0.09 to 0.67)	0.011
DHDL240	0.01 (−0.14 to 0.12)	−0.19 (−0.33 to −0.07)	−0.15 (−0.20 to −0.10)	0.002
DLDL240	−0.11 (−0.40 to 0.13)	−0.58 (−0.86 to −0.38)	−0.55 (−1.00 to −0.38)	0.013
DTC360	−0.07 (−0.63 to 0.62)	−0.56 (−0.96 to −0.24)	−0.12 (−0.36 to 0.05)	0.275
DTG360	−0.09 (−0.19 to 0.66)	0.22 (0.01 to 0.57)	0.26 (−0.09 to 1.04)	0.453
DHDL360	−0.03 (−0.14 to 0.08)	−0.20 (−0.33 to −0.08)	−0.10 (−0.16 to 0.04)	0.042
DLDL360	−0.16 (−0.71 to 0.38)	−0.61 (−0.78 to −0.21)	−0.38 (−0.43 to −0.01)	0.158
DTC480	−0.06 (−0.40 to 0.53)	−0.48 (−0.98 to 0.01)	−0.06 (−0.52 to 0.40)	0.012
DTG480	0.13 (−0.12 to 0.41)	0.18 (−0.26 to 0.70)	0.28 (−0.02 to 0.48)	0.916
DHDL480	−0.04 (−0.23 to 0.03)	−0.14 (−0.24 to −0.01)	−0.13 (−0.28 to −0.10)	0.305
ΔLDL48	0.20 (−0.16 to 0.88)	−0.46 (−0.68 to −0.08)	−0.20 (−0.76 to −0.04)	< 0.001

Results in the table are expressed as median (Q1-Q3) for quantitative data.

For continuous variables, the Kruskal–Wallis rank sum test was used.

All variables in “Characteristics” represent the differences between various time points and the baseline.

For example, DTC120 refers to the difference in TC between week 12 and the baseline, and DTG120 refers to the difference in TG between week 12 and the baseline.

NAs, nucleoside (nucleotide) analogs; TC, total cholesterol; TG, triglycerides; HDL, high-density lipoprotein; LDL, low-density lipoprotein.

### 3.3 Univariate analysis of factors associated with serum ΔLDL48

Univariate analysis showed that age, baseline waist circumference, BMI, treatment regimen, baseline ALT, and HBeAg status were significantly associated with serum ΔLDL48 (*P* < 0.05; Table 3). However, baseline waist circumference was not statistically significant in the multivariate analysis and thus was not included in the final model.

**Table 3:** Univariate analysis for ΔLDL48.

Variables	Statistics	ΔLDL48
Age (years)	42.77 ± 9.16	0.01 (0.00, 0.03) 0.0442
Gender, *n* (%)		
Male	428 (73.04%)	Reference
Female	158 (26.96%)	0.26 (−0.09, 0.60) 0.1511
Waistline (cm)	88.93 ± 10.40	−0.01 (−0.03, −0.00) 0.0485
Height (cm)	1.71 ± 0.08	0.54 (−1.56, 2.64) 0.6155
Weight (kg)	74.15 ± 13.72	−0.01 (−0.02, 0.00) 0.0710
BMI (kg/m^2^)	25.35 ± 3.65	−0.04 (−0.08, −0.01) 0.0185
CHB family history, *n* (%)		
No	227 (44.51%)	Reference
Yes	283 (55.49%)	−0.13 (−0.49, 0.24) 0.4991
With other diseases		
No	257 (85.38%)	Reference
Yes	44 (14.62%)	−0.24 (−0.65, 0.17) 0.2603
Pr-treatment history		
No	143 (47.67%)	Reference
Yes	156 (52.00%)	0.32 (−0.20, 0.85) 0.2343
Treatment grouping		
NAs	96 (16.41%)	Reference
NAs + Peg-IFNalpha-2b	396 (67.69%)	−0.78 (−1.10, −0.46) < 0.0001
Peg-IFNalpha-2b	93 (15.90%)	−0.69 (−1.25, −0.13) 0.0178
Fatty liver, *n* (%)		
No	325 (57.12%)	Reference
Yes	244 (42.88%)	−0.19 (−0.51, 0.12) 0.2307
Cirrhosis		
No	390 (81.25%)	Reference
Yes	90 (18.75%)	0.04 (−0.36, 0.43) 0.8527
HBV DNA (log_10_ IU/ml)	2.94 ± 2.06	−0.05 (−0.13, 0.03) 0.1956
HBV DNA		
Negative	319 (54.72%)	Reference
Positive	264 (45.28%)	−0.05 (−0.37, 0.28) 0.7829
HBsAg (log_10_ IU/ml)	2.92 ± 1.14	−0.04 (−0.20, 0.11) 0.5966
HBeAg		
No	200 (72.46%)	Reference
Yes	76 (27.54%)	0.62 (0.19, 1.06) 0.0077
ALT (U/L)	43.03 ± 53.71	−0.00 (−0.01, −0.00) 0.0417
AST (U/L)	32.35 ± 31.99	−0.00 (−0.01, 0.00) 0.0854
TBIL (μmol/L)	16.77 ± 6.88	0.01 (−0.01, 0.03) 0.3462
ALP (U/L)	74.32 ± 21.21	0.00 (−0.01, 0.01) 0.5635
GGT (U/L)	35.28 ± 58.92	−0.01 (−0.02, 0.00) 0.2187
AFP	6.27 ± 35.86	−0.10 (−0.14, −0.06) < 0.0001
TC at 0 week (mmol/L)	5.77 ± 14.74	−0.25 (−0.41, −0.09) 0.0032
TG at 0 week (mmol/L)	1.45 ± 2.05	−0.07 (−0.30, 0.17) 0.5799
HDL at 0 week (mmol/L)	1.10 ± 0.26	0.27 (−0.43, 0.97) 0.4520

The data in the table is presented as: β (95% CI) *P*-value.

CI, confidence interval. ΔLDL48 refers to the difference in LDL between week 48 and the baseline.

BMI, Body Mass Index; CHB, chronic hepatitis B; NAs, nucleoside (nucleotide) analogs; HBV DNA, hepatitis B virus deoxyribonucleic acid; HBsAg, hepatitis B surface antigen; HBeAg, hepatitis B e antigen; ALT, alanine aminotransferase; AST, aspartate aminotransferase; TBIL, total bilirubin; ALP, alkaline phosphatase; GGT, gamma-glutamyl transferase; AFP, alpha-fetoprotein; TC, total cholesterol; TG, triglycerides; HDL, high-density lipoprotein.

### 3.4 Stratified analysis of the effect of different antiviral regimens on serum ΔLDL48

Stratified analysis revealed that compared with the NAs group, the Peg-IFNalpha-2b + NAs group exhibited significant effects on serum ΔLDL48 across multiple subgroups, including age (>38 years), sex, comorbidities, fatty liver status, cirrhosis status, HBV DNA status, and HBeAg status (*P* < 0.05; Table 4). In the overweight/obese subgroup (BMI ≥28, *n* = 42), the combination group showed an apparent increase in LDL relative to NAs (β = 1.02). This anomalous result is most likely explained by the very small sample size in this subgroup, which leads to increased random variability. This finding should be interpreted as a chance finding due to limited statistical power in this small stratum.

**Table 4:** Stratification analysis of different treatment grouping on ΔLDL48.

Variables	NAs	NAs + Peg-IFNalpha-2b	*P*	Peg-IFNalpha-2b	*P*
Gender					
Male	Reference	−0.84 (−1.27, −0.41)	0.0003	−0.66 (−1.41, 0.08)	0.0848
Female	Reference	−0.61 (−0.96, −0.25)	0.0030	−0.74 (−1.38, −0.11)	0.0324
Age (years) group					
21–38	Reference	−0.40 (−1.00, 0.20)	0.2048	−0.72 (−1.74, 0.29)	0.1751
38–45	Reference	−0.96 (−1.81, −0.11)	0.0408	−0.69 (−2.07, 0.69)	0.3404
46–68	Reference	−0.95 (−1.35, −0.56)	< 0.0001	−0.60 (−1.30, 0.09)	0.0987
BMI					
18.5–23.9	Reference	−0.45 (−1.19, 0.28)	0.2359	−0.17 (−1.15, 0.82)	0.7446
24.0–27.9	Reference	−0.31 (−0.73, 0.11)	0.1562	−0.07 (−0.73, 0.59)	0.8436
≥28.0	Reference	1.02 (−0.12, 2.15)	0.0886	0.02 (−2.14, 2.19)	0.9841
CHB family history					
No	Reference	−17.89 (−35.46, −0.32)	0.0558	−3.54 (−51.99, 44.91)	0.8872
Yes	Reference	−41.11 (−60.34, −21.88)	0.0002	−37.69 (−66.21, −9.16)	0.0138
Other diseases					
No	Reference	−0.66 (−1.04, −0.28)	0.0015	−0.83 (−1.45, −0.21) 0	0.0116
Yes	Reference	−0.98 (−1.55, −0.41)	0.0063	0.08 (−0.91, 1.08)	0.8729
Pr-treatment history					
No	Reference	−1.26 (−2.83, 0.32)	0.1445	−0.84 (−2.58, 0.91)	0.3658
Yes	Reference	−0.66 (−1.19, −0.12)	0.0226	−0.73 (−1.02, 0.12)	0.0748
Fatty liver, *n* (%)					
No	Reference	−0.75 (−1.07, −0.42)	< 0.0001	−0.66 (−1.28, −0.05)	0.0408
Yes	Reference	−0.82 (−1.39, −0.24)	0.0082	−0.69 (−1.62, 0.25)	0.1585
Cirrhosis					
No	Reference	−0.92 (−1.35, −0.49)	< 0.0001	−0.77 (−1.50, −0.03)	0.0454
Yes	Reference	−0.52 (−1.01, −0.04)	0.0500	−0.29 (−1.31, 0.73)	0.5847
HBV DNA					
Negative	Reference	−0.76 (−1.07, −0.44)	< 0.0001	−0.37 (−1.16, 0.42)	0.3576
Positive	Reference	−1.07 (−1.77, −0.38)	0.0053	−1.07 (−1.99, −0.14)	0.0315
HBeAg					
Negative	Reference	−0.65 (−1.28, −0.03)	0.0493	−0.82 (−1.61, −0.03)	0.0515
Positive	Reference	−0.93 (−1.40, −0.46)	0.0030	−0.96 (−1.46, −0.52)	0.0041
ALT (U/L)					
18–40	Reference	−27.56 (−39.32, −15.80)	< 0.0001	−24.38 (−46.85, −1.90)	0.0370
>40	Reference	−38.09 (−92.71, 16.53)	0.2049	−38.25 (−107.34, 30.84)	0.3061

The data in the table is presented as: β (95% CI) *P*-value.

ΔLDL48 refers to the difference in LDL between week 48 and the baseline.

NAs, nucleoside (nucleotide) analogs; BMI, Body Mass Index; CHB, chronic hepatitis B; HBV DNA, hepatitis B virus deoxyribonucleic acid; HBeAg, hepatitis B e antigen; ALT, alanine aminotransferase.

### 3.5 Multivariable regression models of the effect of different antiviral regimens on serum LDL

Across unadjusted models, models adjusted for sex and age, and models adjusted for sex, age, BMI, and fatty liver, treatment regimens containing Peg-IFNalpha-2b (Peg-IFNalpha-2b monotherapy or Peg-IFNalpha-2b + NAs) consistently showed significant LDL-lowering effects compared with NAs therapy alone (*P* < 0.05; Table 5).

**Table 5:** Relationship between different treatment grouping and ΔLDL48 in different models.

Variable	Crude model		Model I		Model II	
	β (95% CI)	*P*-value	β (95% CI)	*P*-value	β (95% CI)	*P*-value
Treatment grouping						
NAs	Reference		Reference		Reference	
NAs + Peg-IFNalpha-2b	−0.8 (−1.1, −0.5)	< 0.001	−0.7 (−1.1, −0.4)	< 0.001	−0.67 (−1.01, −0.33)	< 0.001
Peg-IFNalpha-2b	−0.7 (−1.2, −0.1)	0.018	−0.6 (−1.2, −0.1)	0.026	−0.58 (−1.14, −0.01)	0.049

Generalized additive mixed models were used to adjust for key confounders.

ΔLDL48 refers to the difference in LDL between week 48 and the baseline.

Crude model adjust for: none.

Model I adjust for: gender, age.

Model II adjust for: gender, age, BMI, previous treatment history, fatty liver, cirrhosis, HBV DNA, HBsAg, HBeAg, LDL, TBIL, ALP at baseline.

CI, confidence interval. NAs, nucleoside (nucleotide) analogs.

### 3.6 Longitudinal changes in HBsAg levels during follow-up

**Figure 5:**
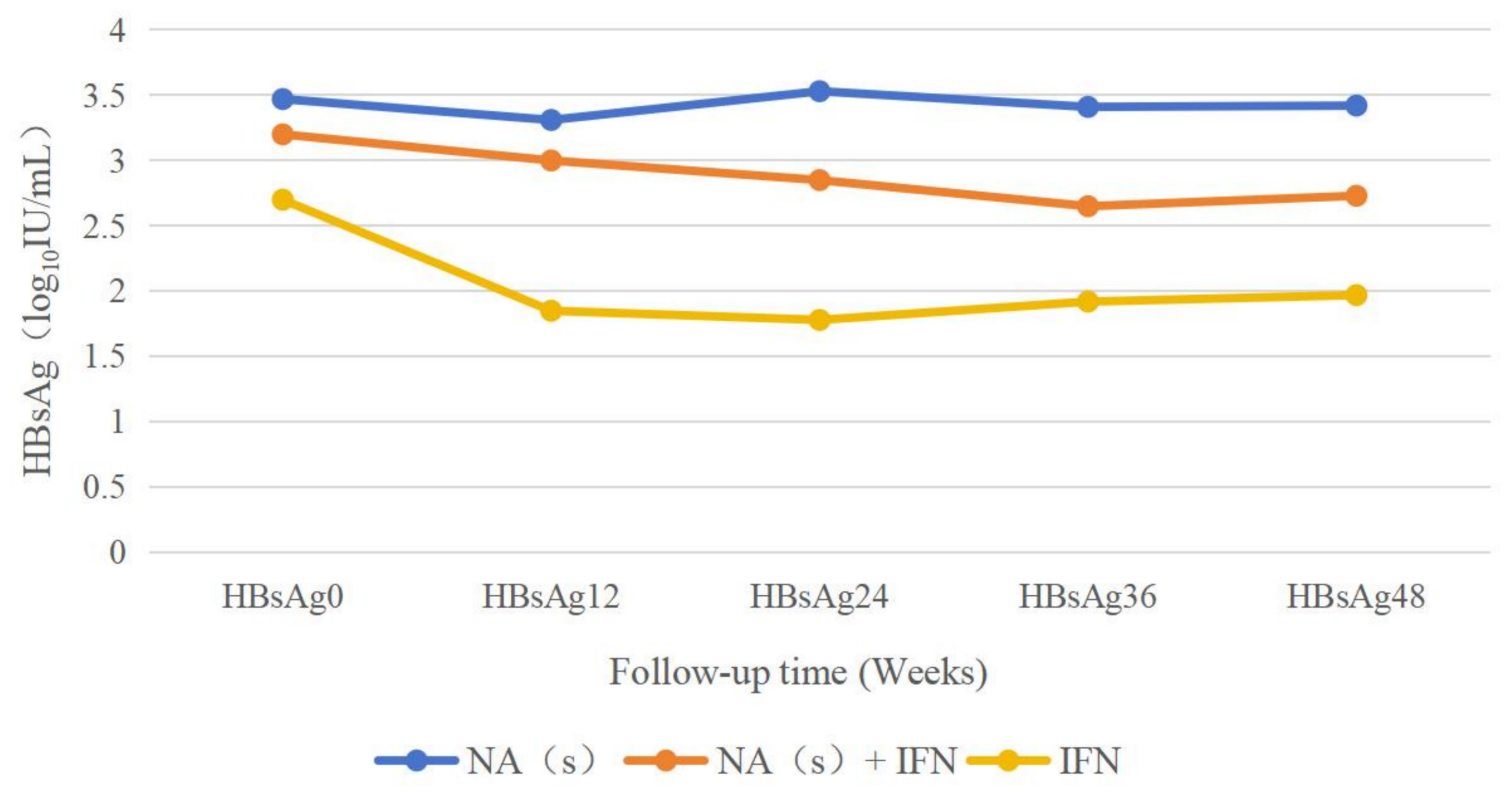
Longitudinal dynamic changes of serum HBsAg levels during 48-week follow-up across antiviral regimens. This figure depicts 48-week dynamic HBsAg changes across three treatment groups: NAs monotherapy, NAs + IFN combination, and IFN monotherapy. Normality tests confirmed non-normal HBsAg distribution across all time points, thus median values were adopted for plotting. The x-axis indicates follow-up time points (baseline, week 12/24/36/48); the y-axis represents log10-transformed HBsAg levels (IU/ml). Colored lines represent group-specific trends, which correspond to the concurrent dynamic change trend of LDL levels.

During the 48-week follow-up, distinct dynamic trends were observed: the NA monotherapy group maintained stable HBsAg levels throughout treatment, while both Peg-IFNalpha-2b-containing groups showed a gradual, sustained reduction. At week 48, the median HBsAg levels reached 3.42 (2.73 to 3.65) log10 IU/ml in the NA group, 2.73 (1.35 to 3.42) IU/ml in the combination group, and 1.97 (−0.47 to 3.03) IU/ml in the Peg-IFNalpha-2b group, with a statistically significant between-group difference (*P* < 0.001). We have added a new line graph (Figure 5) to visually display the dynamic trend of HBsAg levels in the three groups during follow-up.

## 4 Discussion

This prospective analysis of 585 patients with CHB found that treatment with Peg-IFNalpha-2b significantly affected serum LDL levels. Specifically, in both the NAs + Peg-IFNalpha-2b group and the Peg-IFNalpha-2b monotherapy group, after 48 weeks of treatment, ΔLDL48 was significantly lower compared with the NAs group (*P* < 0.05). This difference remained statistically significant after adjusting for sex, age, BMI, and fatty liver status (*P* < 0.05). Univariate analysis showed that treatment regimen was an independent factor influencing LDL change. Stratified analysis demonstrated that this effect was consistent across subgroups stratified.

The proportion of combination therapy in this study was relatively high (67.69%), primarily attributed to the inclusion of NAs-experienced patients pursuing clinical cure rather than treatment-naïve individuals. According to the EASL 2025 Clinical Practice Guidelines, adding pegylated interferon α (Peg-IFNα) to this specific patient cohort can enhance the hepatitis B surface antigen (HBsAg) clearance rate (12). This is consistent with our study finding that 45 patients (9.20%) in the combination therapy group achieved HBsAg clearance, thereby validating the clinical applicability of this treatment regimen.

The high MASLD prevalence (58.63%) in our study population is attributable to the high proportion of overweight patients (mean BMI 25.35 ± 3.65 kg/m^2^) and the close association between chronic HBV infection and metabolic syndrome. Previous studies have shown that HBV infection may interfere with hepatic lipid metabolism, increasing the risk of NAFLD (13). Beyond this, chronic HBV infection itself is well-documented to be associated with lower total cholesterol and LDL-C levels compared with the general population, a well-recognized consensus consistently confirmed by multiple large-scale clinical cohorts (14, 15), which aligns with the baseline lipid findings of our own study. This hypolipidemic effect arises from two key factors: HBV's direct disruption of host hepatic lipid synthesis pathways, and progressive impairment of liver synthetic function in advanced liver disease, which further reduces circulating lipid levels (15, 16).

Notably, first-line nucleos(t)ide analogs exert distinct impacts on lipid profiles: TDF has a mild LDL-lowering effect, TAF links to relatively higher lipids, while ETV has a neutral serum lipid effect (17–20). To rule out NA-related confounding, we analyzed baseline NA distribution. As shown in Table 1, ETV/TDF/TAF proportions were highly balanced: 50.0, 30.2, 19.8% in the NA group, vs. 44.2, 34.1, 21.7% in the combination group, with no significant difference (*P* = 0.122). This confirmed the LDL reduction in the Peg-IFNalpha-2b group was independent of baseline NA treatment, eliminating NA-related lipid changes as a confounder, ensuring our findings are not biased by baseline antiviral regimen differences.

In this study, we took several steps to minimize the impact of confounding factors on our results. We ensured well-balanced baseline characteristics (age, gender, BMI, fatty liver prevalence) across all three treatment groups. We further adjusted for these key confounders in progressive multivariate regression models, and the association between Peg-IFNalpha-2b treatment and LDL reduction remained statistically significant across all models. Residual confounding remains a potential limitation: healthier lifestyle modifications in the interferon group could overestimate the effect. However, the result strongly supports that the observed LDL reduction is primarily attributable to Peg-IFNalpha-2b treatment. Future prospective randomized trials with standardized lifestyle documentation will further validate these findings. In univariate analysis, AFP levels showed a significant inverse association with ΔLDL48 (β = −0.10, *P* < 0.0001). While this may reflect a statistical artifact due to multiple testing, a potential biological explanation is that higher AFP levels (indicating more active hepatic inflammation) may be associated with greater interferon-induced metabolic modulation. This finding should be interpreted cautiously and requires validation in future studies.

Previous studies have shown that HBV infection is closely associated with lipid metabolism disorders. HBV infection may lead to elevated LDL levels, whereas PEG-IFN treatment can effectively reduce LDL and help restore normal lipid metabolism (7), consistent with the findings of this study. This may be related to PEG-IFN's ability to improve hepatic inflammation, promote hepatocyte regeneration, and enhance hepatic lipid metabolism. Current evidence suggests that in chronic HBV patients, PEG-IFN therapy not only facilitates partial viral clearance but also improves lipid metabolism indicators—specifically lowering LDL and raising HDL (21). The LDL-lowering effect of Peg-IFNα-2b is driven by two synergistic molecular mechanisms. First, Peg-IFNα-2b acts via the canonical JAK-STAT signaling pathway (22) to transcriptionally upregulate hepatic low-density lipoprotein receptor (LDLR) expression, enhancing circulating LDL particle clearance (23). Second, interferon signaling directly induces proteasomal degradation of HMGCR, the rate-limiting enzyme for endogenous cholesterol biosynthesis, inhibiting *de novo* cholesterol production (24). These complementary mechanisms collectively underpin the sustained, clinically meaningful LDL reduction observed in our CHB patient cohort.

Other research also demonstrates that during PEG-IFN treatment, LDL levels decrease significantly. In a randomized controlled trial involving 253 patients, the Peg-IFNalpha-2b group showed a significant reduction in LDL compared with controls, and this reduction correlated with HBsAg clearance rates, suggesting PEG-IFN may enhance its therapeutic effect by improving lipid metabolism (25). However, in our study, when we stratified patients in the Peg-IFNalpha-2b-containing groups into HBsAg clearance and non-clearance groups, logistic regression analysis confirmed that the 48-week LDL reduction amplitude was not an independent factor associated with HBsAg clearance.

Notably, our study observed a dynamic biphasic change in LDL levels among patients receiving Peg-IFNalpha-2b-containing regimens: while a statistically significant reduction in LDL was achieved at the 48-week endpoint, a mild transient rebound in LDL levels was noted between weeks 12 and 36 in both the Peg-IFNalpha-2b monotherapy and combination therapy groups. This transient fluctuation might reflect interindividual differences in metabolic adaptation to interferon therapy, which normalized by week 48 in our cohort. More importantly, this dynamic pattern of LDL changes during long-term Peg-IFNalpha-2b treatment provides a critical mechanistic explanation for the inconsistent findings across previous clinical studies.

In this study the mild rebound in LDL levels at weeks 12–36 in the Peg-IFNalpha-2b group might reflect interindividual differences in metabolic adaptation to interferon therapy, which normalized by week 48 in our cohort. These discrepancies may stem from the following reasons: first, differences in baseline characteristics of study populations. In this study, baseline LDL levels in the NAs + Peg-IFNalpha-2b group (2.79 ± 0.76 mmol/L) were significantly higher than those in the NAs group (2.49 ± 0.58 mmol/L), whereas some other studies included more CHB patients with normal baseline lipid levels. Second, differences in treatment regimens. In this study, the Peg-IFNalpha-2b dosage was 180 μg/week, while some studies used lower doses (e.g., 135 μg/week) (26). Third, differences in follow-up duration. This study followed patients for 48 weeks, which could observe the cumulative effect of long-term treatment on LDL. Fourth, differences in statistical methods. This study employed a generalized additive mixed model to adjust for multiple confounding factors.

This study has several key strengths addressing critical gaps in previous research: first, it is one of the relatively large sample size of real-world studies, enrolling 585 CHB patients—significantly larger than most prior studies (typically < 150 patients). The rigorous three-group design (NAs: 96; NAs + Peg-IFNalpha-2b: 396; Peg-IFNalpha-2b: 93) enables direct comparison of lipid changes across all major antiviral regimens, reducing sampling error and improving generalizability. Second, we performed a complete 48-week longitudinal assessment with monitoring at four time points (weeks 12, 24, 36, 48), capturing dynamic lipid changes that most prior studies (limited to baseline and end-of-treatment measurements) missed. Third, we rigorously adjusted for key confounders (Gender, age, BMI, Previous treatment history, Fatty liver, Cirrhosis, HBV DNA, HBsAg, HBeAg, LDL, TBIL, ALP at baseline) in multivariate models. Fatty liver, a critical lipid confounder often overlooked in prior work, was explicitly controlled for, enhancing result reliability. Fourth, consistent findings across multiple subgroups (age, gender, fatty liver, baseline HBsAg) confirm result robustness, a validation rarely reported in smaller studies.

Nevertheless, this study also has some limitations: first, as a real-world cohort study, it is subject to selection bias. Nevertheless, this study has several limitations. First, as a real-world cohort study, it is subject to selection bias: patients in the NAs + Peg-IFNα-2b group had better baseline viral suppression, and treatment allocation was not randomized. Second, LDL subfractions (e.g., small dense LDL) and advanced lipid markers [e.g., lipoprotein (a)] were not assessed, limiting comprehensive evaluation of Peg-IFNα-2b's effects on lipid metabolism (27). Third, residual confounding remains: although we adjusted for key confounders including sex, age, BMI, and MASLD status, we lacked detailed data on dietary patterns, physical activity, insulin resistance, and socioeconomic status, which may influence lipid levels (28). All patients received standardized lifestyle education at baseline, but longitudinal lifestyle records during treatment were not collected. Fourth, this study presents 48-week interim results; post-treatment lipid follow-up is ongoing and full long-term data will be published separately. Fifth, 36 patients discontinued Peg-IFNα-2b due to intolerance, introducing potential survivorship bias. However, our sensitivity analysis confirmed no significant baseline differences between discontinuers and completers, and the LDL-lowering effect remained robust after conservative adjustment. Finally, the Peg-IFNα-2b product used (Pegbing^®^, Xiamen Amoytop Biotech) is a Chinese domestic biosimilar; caution is warranted when generalizing findings to other global formulations due to potential pharmacokinetic differences.

LDL metabolism is closely associated with the progression of various liver diseases. In the context of MASLD or CHB combined with MASLD, elevated LDL serves as a key risk factor for disease progression: it is closely linked to insulin resistance, exacerbated hepatic fat accumulation, and pro-inflammatory/pro-fibrotic states, which can accelerate liver fibrosis (29). Comorbid MASLD also worsens fibrosis in CHB patients, acting as an independent predictor of fibrosis progression (30). Beyond this, oxidized LDL can directly drive disease development by activating hepatic stellate cells and inducing oxidative stress and inflammatory responses (31). In CHB patients, LDL levels also correlate with disease severity, with further reductions observed as the disease progresses to cirrhosis (32).

Beyond hepatic implications, LDL reduction also confers well-validated cardiovascular benefits. The 0.3–0.4 mmol/L LDL reduction observed in our study is clinically meaningful, as the landmark CTT Collaboration demonstrated a linear relationship between LDL reduction and cardiovascular risk reduction in statin trials (33). While direct extrapolation to interferon therapy is not appropriate, this magnitude of LDL reduction is associated with clinically relevant cardiovascular benefit in large patient cohorts. Modulating LDL metabolism is therefore an effective clinical strategy: statin use can lower LDL and attenuate hepatic injury (34), while the LDL reduction induced by Peg-IFNα-2b may also bring potential long-term dual hepatic and cardiovascular benefits, especially for patients with comorbid metabolic disorders.

This study demonstrates that Peg-IFNalpha-2b treatment in CHB patients can significantly reduce serum LDL levels, and that this effect persists when combined with NAs therapy. These findings suggest that, for patients pursuing clinical cure who have elevated baseline LDL levels or concomitant cardiovascular risk factors, the dual benefit of LDL reduction and HBsAg clearance from Peg-IFNalpha-2b therapy should be carefully considered. It is noteworthy, however, that these results apply only to CHB patients receiving first-line NAs (ETV/TDF/TAF) and/or Peg-IFNalpha-2b, and cannot be extrapolated to other antiviral regimens or liver disease populations. Moreover, since this study did not directly assess whether LDL reduction translates into fewer cardiovascular events, future long-term follow-up studies are needed to clarify the prognostic implications of Peg-IFNalpha-2b–induced LDL reduction.

## Data Availability

The raw data supporting the conclusions of this article will be made available by the authors, without undue reservation.
